# Vital Signs: Racial Disparities in Age-Specific Mortality Among Blacks or African Americans — United States, 1999–2015

**DOI:** 10.15585/mmwr.mm6617e1

**Published:** 2017-05-05

**Authors:** Timothy J. Cunningham, Janet B. Croft, Yong Liu, Hua Lu, Paul I. Eke, Wayne H. Giles

**Affiliations:** ^1^Division of Population Health; ^2^Division of Heart Disease and Stroke Prevention, National Center for Chronic Disease Prevention and Health Promotion, CDC.

## Abstract

**Background:**

Although the overall life expectancy at birth has increased for both blacks and whites and the gap between these populations has narrowed, disparities in life expectancy and the leading causes of death for blacks compared with whites in the United States remain substantial. Understanding how factors that influence these disparities vary across the life span might enhance the targeting of appropriate interventions.

**Methods:**

Trends during 1999–2015 in mortality rates for the leading causes of death were examined by black and white race and age group. Multiple 2014 and 2015 national data sources were analyzed to compare blacks with whites in selected age groups by sociodemographic characteristics, self-reported health behaviors, health-related quality of life indicators, use of health services, and chronic conditions.

**Results:**

During 1999–2015, age-adjusted death rates decreased significantly in both populations, with rates declining more sharply among blacks for most leading causes of death. Thus, the disparity gap in all-cause mortality rates narrowed from 33% in 1999 to 16% in 2015. However, during 2015, blacks still had higher death rates than whites for all-cause mortality in all groups aged <65 years. Compared with whites, blacks in age groups <65 years had higher levels of some self-reported risk factors and chronic diseases and mortality from cardiovascular diseases and cancer, diseases that are most common among persons aged ≥65 years.

**Conclusions and Implications for Public Health Practice:**

To continue to reduce the gap in health disparities, these findings suggest an ongoing need for universal and targeted interventions that address the leading causes of deaths among blacks (especially cardiovascular disease and cancer and their risk factors) across the life span and create equal opportunities for health.

## Introduction

Blacks or African Americans (referred to as blacks in this report) are the third largest racial/ethnic population in the United States, after whites and Hispanics (*1*). In 2014, life expectancy at birth was 75.6 years for blacks and 79.0 years for whites, an increase of 3.8 years from 71.8 years and an increase of 1.7 years from 77.3 years in 2000, respectively (*2*). Despite this improvement, disparities in the leading causes of deaths for blacks compared with whites are pronounced by early and middle adulthood, especially deaths from homicide and chronic conditions such as heart disease and diabetes (*2*,*3*). In addition, blacks have the highest death rate and shorter survival rate for all cancers combined compared with whites in the United States (*4*). Although many of these chronic conditions are usually associated with adulthood, the initial stages of some chronic conditions arise early in life (*5*). The analyses presented in this report used recent mortality and two national surveillance data sets to compare rates for the leading causes of death and the prevalences of chronic diseases, related health behaviors, health-related quality of life indicators, and health care utilization practices for blacks compared with whites by age group to identify disparities across the life span; such information could facilitate targeted interventions.

## Methods

Age-adjusted death rates for blacks and whites of all ages (including children) and also age-specific trends for the leading selected causes of death among blacks for four adult age groups (18–34, 35–49, 50–64, and ≥65 years) were examined for the period 1999–2015. In addition, age-specific sociodemographic characteristics and death rates were examined and compared by race and age group. Age-specific prevalences of selected self-reported chronic diseases, related health behaviors, health-related quality of life indicators, and health care utilization practices were also examined and compared by race and age group.

Mortality data were analyzed using the CDC WONDER system, an interactive Web-based tool.* CDC WONDER mortality data are provided by the National Vital Statistics System and are based on information from all resident death certificates filed in the 50 states and the District of Columbia. CDC WONDER queries generated age-specific death rates and 95% confidence intervals for blacks and whites for all causes of death and leading causes of death among blacks^†^ compared with whites in each age group during 1999–2015. Age-adjusted death rates also were obtained for all ages combined, including children. Rate ratios compared death rates for blacks to those for whites; the 95% confidence interval (CI) for each rate ratio was calculated (*6*), and statistical significance was determined at alpha = 0.05; 95% CIs that did not include 1.0 were considered indicative of a statistically significant difference between blacks and whites.

Population numbers, the sex distribution, and the percentage of each race with a Hispanic origin were obtained for each age group from the most recent available estimated postcensal population counts for 2014^§^ from the U.S. Census Bureau. Selected socioeconomic characteristics (U.S. nativity, <12 years education, household poverty, home ownership by the household head, and lack of health insurance) of the 2014 population by race and age group were obtained from the 2014 American Community Survey Public Use Microdata Sample,^¶^ which is an ongoing national household survey of the U.S. Census Bureau.

Self-reported information on chronic diseases, health behaviors, health-related quality of life indicators, and health care utilization practices were obtained from the 2015 Behavioral Risk Factor Surveillance System (BRFSS), which is an annual state-based, random-digit-dialed telephone (cell phone and landline) survey of the noninstitutionalized U.S. population aged ≥18 years.** The median state response rate for the combined landline and cell phone surveys was 47.2%. Self-reported health behaviors among all respondents included current cigarette smoking (having smoked at least 100 cigarettes in the lifetime and smoking daily or somedays), lack of leisure-time physical activity in the past 30 days, and binge drinking (five or more drinks for men, or four or more drinks for women on any occasion) in the past 30 days. Weight status indicators included having a normal body weight (body mass index of 18.5–24.9 kg/m^2^), and having obesity (body mass index ≥30 kg/m^2^) based on self-reported height and weight. Health care access and utilization indicators included having a personal doctor or health care provider, not being able to see a doctor in the past year because of cost, and taking medication to control high blood pressure (among adults with high blood pressure). Self-reported health-related quality of life indicators included fair or poor health status, frequent mental distress (≥14 days in past 30 days), and frequent physical distress (≥14 days in past 30 days). Chronic disease conditions included reporting ever having been told by a doctor or other health professional that the respondent had asthma, chronic obstructive pulmonary disease, high blood pressure, high blood cholesterol, diabetes, coronary heart disease (including heart attack or angina), stroke, or cancer (excluding skin cancer).

Statistical software that accounts for the complex sampling design of the BRFSS was used for analyses to obtain age-specific prevalences by race, prevalence ratios that compared blacks with whites, and CIs. For comparisons of BRFSS indicators by race, statistical significance (p<0.05) was determined in age-specific logistic regression by the Wald F-test.

## Results

**FIGURE 1:**
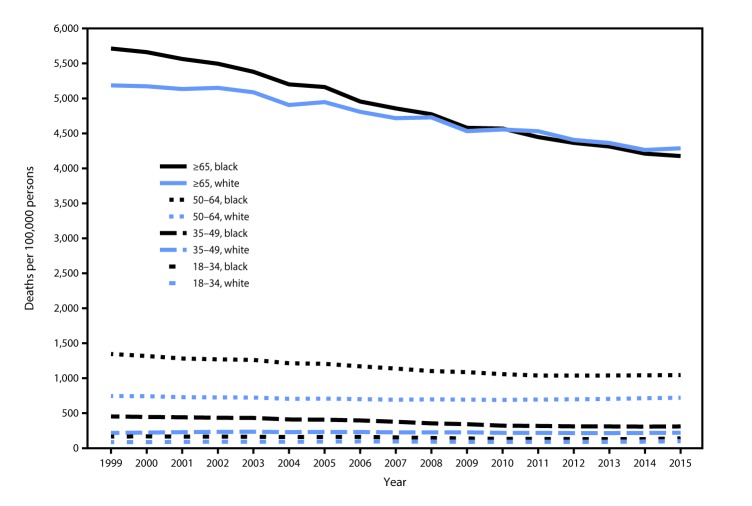
Death rates among blacks and whites, by age group (years) — United States, 1999–2015

In 1999, age-adjusted death rates for any cause of death were 1,135.7 per 100,000 blacks and 854.6 per 100,000 whites (Table 1). By 2015, the racial gap had narrowed with age-adjusted death rates of 851.9 per 100,000 blacks and 735.0 per 100,000 whites. The age-adjusted death rate in 2015 relative to that in 1999 had declined 25% for blacks and 14% for whites; there were 284 fewer age-adjusted deaths per 100,000 blacks during 2015 compared with 1999, whereas there were 120 fewer age-adjusted deaths per 100,000 whites. The disparity gap in all-cause mortality rates decreased from 33% in 1999 to 16% in 2015. Among adults aged ≥65 years, the death rate in 2015 relative to that in 1999 declined 27% for blacks and 17% for whites, resulting in a crossover in death rates beginning in 2010, when blacks had lower death rates than whites (Figure 1).

**TABLE 1:** Death rates per 100,000 population for selected leading causes of death, percentage changes, and death rate disparities between blacks and whites, by age group — National Vital Statistics System, United States, 1999 and 2015

Cause of death by age group (yrs)	Blacks			Whites			Death rate disparity relative to whites*	
	1999 rate	2015 rate	% change (1999 to 2015)	1999 rate	2015 rate	% change (1999 to 2015)	1999 (%)	2015 (%)
**1. All causes**								
All ages^†^	1,135.7	851.9	-25.0^§^	854.6	735.0	-14.1^§^	+32.9^§^	+15.9^§^
18–34	167.8	141.5	-15.6^§^	87.5	100.3	+14.6^§^	+91.8^§^	+41.1^§^
35–49	454.3	311.5	-31.4^§^	218.2	220.3	+1.0^§^	+108.2^§^	+41.4^§^
50–64	1,346.5	1,046.0	-22.3^§^	746.5	722.4	-3.2^§^	+80.4^§^	+44.8^§^
≥65	5,712.7	4,176.0	-26.9^§^	5,186.0	4,286.9	-17.3^§^	+10.2^§^	-2.6^§^
**2. Diseases of the heart**								
All ages	334.3	205.1	-38.7^§^	262.0	167.9	-35.9^§^	+27.6^§^	+22.2^§^
18–34	12.5	10.7	-14.5^§^	4.8	5.1	+5.2	+158.7^§^	+110.3^§^
35–49	85.3	66.5	-22.0^§^	37.9	33.3	-12.0^§^	+125.2^§^	+99.7^§^
50–64	378.6	257.5	-32.0^§^	193.9	148.1	-23.6^§^	+95.2^§^	+73.9^§^
≥65	1,902.6	1,085.5	-42.9^§^	1,756.7	1,091.8	-37.9^§^	+8.3^§^	-0.6
**3. Malignant neoplasms**								
All ages	252.5	180.1	-28.7^§^	198.0	159.4	-19.4^§^	+27.6^§^	+13.0^§^
18–34	9.6	7.3	-23.8^§^	7.8	6.4	-17.4^§^	+22.7^§^	+13.2^§^
35–49	79.8	49.2	-38.3^§^	51.6	39.9	-22.6^§^	+54.6^§^	+23.2^§^
50–64	408.2	296.2	-27.4^§^	280.4	227.6	-18.8^§^	+45.6^§^	+30.1^§^
≥65	1,305.0	927.7	-28.9^§^	1,118.8	893.9	-20.1^§^	+16.6^§^	+3.8^§^
**4. Cerebrovascular diseases**								
All ages	81.8	50.8	-37.9^§^	59.6	36.4	-39.0^§^	+37.4^§^	+39.8^§^
18–34	1.9	1.6	-17.2	1.0	0.8	-15.5^§^	+97.9^§^	+93.9^§^
35–49	20.0	12.8	-36.2^§^	6.0	4.9	-17.4^§^	+236.2^§^	+159.8^§^
50–64	76.8	50.5	-34.3^§^	25.9	21.0	-18.9^§^	+196.5^§^	+140.3^§^
≥65	483.4	287.2	-40.6^§^	426.1	252.5	-40.7^§^	+13.4^§^	+13.8^§^
**5. Unintentional injury**								
All ages	40.1	36.8	-8.0^§^	35.2	46.0	+30.7^§^	+13.8^§^	-20.0^§^
18–34	32.6	30.8	-5.6^§^	34.3	45.4	+32.4^§^	-4.9^§^	-32.1^§^
35–49	43.0	39.5	-8.0^§^	33.5	49.1	+46.8^§^	+28.4^§^	-19.5^§^
50–64	44.6	55.4	+24.4^§^	28.6	50.5	+76.3^§^	+55.7^§^	+9.9^§^
≥65	88.6	69.8	-21.2^§^	94.0	114.4	+21.7^§^	-5.7^§^	-39.0^§^
**6. Diabetes mellitus**								
All ages	49.7	37.0	-25.6^§^	22.6	19.6	-13.3^§^	+120.0^§^	+88.7^§^
18–34	2.1	2.7	+26.9^§^	0.9	1.1	+18.3^§^	+128.0^§^	+144.5^§^
35–49	12.8	13.1	+2.9	5.2	6.2	+19.6^§^	+148.0^§^	+113.3^§^
50–64	69.0	51.8	-24.9^§^	24.7	25.9	+4.8^§^	+179.3^§^	+100.2^§^
≥65	273.0	189.4	-30.6^§^	137.9	110.4	-19.9^§^	+97.9^§^	+71.5^§^
**7. Homicide**								
All ages	20.1	19.8	-1.6	3.8	3.3	-13.0^§^	+434.3^§^	+504.3^§^
18–34	47.6	47.2	-0.8	6.8	5.5	-18.5^§^	+605.0^§^	+758.7^§^
35–49	21.2	21.9	+3.1	4.4	4.2	-5.9^§^	+380.7^§^	+426.5^§^
50–64	9.5	10.1	+6.2	2.7	2.8	+5.3	+255.6^§^	+258.9^§^
≥65	6.6	4.1	-38.0^§^	2.1	1.8	-16.0^§^	+212.7^§^	+130.9^§^
**8. HIV disease**								
All ages	23.6	7.9	-66.8^§^	2.9	1.1	-63.8^§^	+706.8^§^	+641.5^§^
18–34	17.1	3.4	-80.3^§^	2.4	0.4	-84.8^§^	+622.4^§^	+838.9^§^
35–49	56.9	12.2	-78.5^§^	7.5	1.8	-76.5^§^	+657.2^§^	+590.4^§^
50–64	33.2	19.7	-40.7^§^	3.5	2.7	-22.6^§^	+847.7^§^	+625.8^§^
≥65	9.1	10.5	+15.8^§^	0.7	1.1	+55.7^§^	+1,197.1^§^	+864.2^§^
**9. Suicide**								
All ages	5.6	5.6	+0.5	11.3	15.1	+33.8^§^	-50.7^§^	-62.9^§^
18–34	9.5	9.4	-0.4	13.0	16.8	+29.2^§^	-27.1^§^	-43.8^§^
35–49	7.3	7.5	+3.6	15.8	20.8	+31.1^§^	-54.2^§^	-63.8^§^
50–64	4.9	5.5	+13.4	13.8	22.8	+64.6^§^	-64.9^§^	-75.8^§^
≥65	5.7	4.0	-30.0^§^	16.9	18.4	+9.0^§^	-66.4^§^	-78.4^§^

**Abbreviation:** HIV = human immunodeficiency virus.

* Disparity (%) = (Black rate minus white rate) divided by white rate times 100.

^†^ “All ages” category includes infants and children. Death rates for all ages were age-standardized to the 2000 U.S. projected population.

^§^ Z-statistic significant at p<0.05 for the rate change from 1999 to 2015 or for the difference between black and white populations.

**FIGURE 2:**
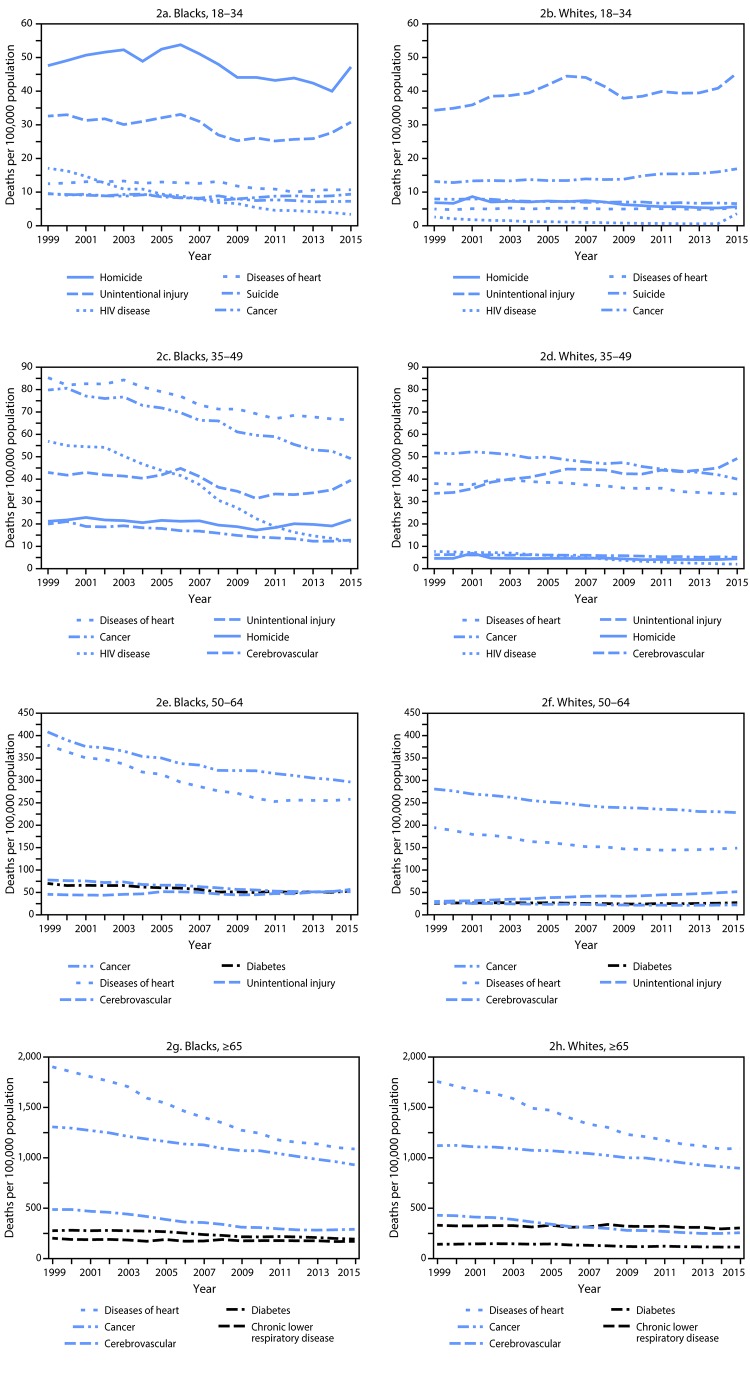
Death rates for selected leading causes of death among black and white adults, by age group (years) — United States, 1999–2015

Age-specific deaths for selected leading causes of death among blacks declined between 1999 and 2015 (Figure 2). Notable declines, for example, included heart disease (15%), cancer (24%), and human immunodeficiency virus (HIV) disease (80%) at ages 18–34 years; heart disease (22%), cancer (38%), and HIV disease (79%) at ages 35–49 years; and heart disease (32% and 43%), cancer (27% and 29%) and cerebrovascular disease (34% and 41%) for the 50–64 and ≥65 age groups (Table 1).

During 2014, sociodemographic characteristics differed by race (Table 2). Blacks in each age group were more likely than whites to have <12 years of education, to be unemployed, live below the poverty level, and less likely to live in a household where the head of household owned the home. Blacks were more likely to have no health insurance than whites for the 18–34, 35–49, and 50–64 age groups, but few persons in either population reported having no health insurance at age ≥65 years.

**TABLE 2:** Selected sociodemographic characteristics of blacks and whites, by age group — U.S. Census Bureau, United States, 2014

Characteristic	All ages		18–34 years		35–49 years		50–64 years		≥65 years	
	Black	White	Black	White	Black	White	Black	White	Black	White
Census population (no. in millions)*	**45.7**	**253.7**	**12.1**	**57.6**	**8.6**	**48.1**	**7.7**	**51.2**	**4.2**	**39.7**
Sex (%)*										
Male	47.9	49.5	49.4	51.2	47.0	50.3	46.0	49.0	40.0	44.4
Female	52.1	50.5	50.6	48.8	53.0	49.7	54.0	51.0	60.0	55.6
**Other characteristics (%)**										
Hispanic*	7.4	19.8	8.0	24.3	6.9	21.6	4.5	12.5	3.6	8.3
U.S.-born^†^	88.4	87.3	87.7	85.0	83.6	81.6	85.6	87.8	87.8	89.8
<12 years education^†^	38.4	29.5	13.8	10.1	11.0	9.9	15.2	9.4	29.4	15.2
Unemployed^†^	6.0	3.0	12.5	6.2	7.6	3.8	5.2	3.1	1.1	0.7
Household below poverty level^†^	25.0	12.2	24.6	15.5	19.4	10.3	19.7	9.2	17.1	7.8
Household head owns home^†^	40.9	65.2	30.7	47.2	42.8	65.5	54.5	77.7	62.9	79.7
No health insurance^†^	13.7	10.1	24.0	17.7	19.3	14.8	13.7	9.8	1.5	0.6

* Number and percentage based on estimated postcensal population counts for 2014 from the U.S. Census Bureau.

^†^ Percentage based on population counts obtained from the Public Use Microdata Sample of the 2014 American Community Survey of the U.S. Census Bureau.

During 2015, health behaviors differed between the two populations (Table 3). Blacks were more likely to be obese, to have no leisure time physical activity, and less likely to have a normal body weight in all age groups compared with whites. In contrast, blacks were less likely to report binge drinking than whites. Although blacks had higher prevalences of current cigarette smoking than whites at ages 50–64 years and ≥65 years, they had a lower prevalence at ages 18–34 years.

**TABLE 3:** Comparison of selected health characteristics reported by black and white adults, by age group and prevalence ratio (PR) — Behavioral Risk Factor Surveillance Survey, United States, 2015

Health characteristic	Adults ≥18 years*		18–34 years		35–49 years		50–64 years		≥65 years	
	Black	White	Black	White	Black	White	Black	White	Black	White
**Unweighted sample size (no.)**	**36,362**	**359,668**	**6,818**	**49,089**	**7,673**	**63,011**	**11,712**	**113,443**	**10,159**	**134,125**
**Estimated noninstitutionalized population (no. in millions)**	**31.1**	**183.7**	**10.7**	**51.2**	**8.0**	**41.9**	**7.8**	**50.0**	**4.6**	**40.6**
***Health behaviors: % (95% CI)***										
**Current smoker**	19.2 (18.5–20.0)	17.5 (17.3–17.8)	19.0 (17.5–20.5)	19.7 (19.1–20.3)	19.8 (18.3–21.3)	19.8 (19.3–20.4)	23.9 (22.4–25.4)	18.4 (18.0–18.8)	11.7 (10.6–12.9)	8.5 (8.2– 8.7)
PR (95% CI)	1.09 (1.05–1.14)^†^	—	0.96 (0.88–1.05)	—	1.00 (0.92–1.08)	—	1.30 (1.21–1.39)^†^	—	1.38 (1.25–1.54)^†^	—
**No leisure-time physical activity in past 30 days**	31.0 (30.1–32.0)	24.6 (24.2–24.9)	25.7 (23.9–27.4)	18.6 (18.0–19.2)	30.2 (28.4–32.1)	24.7 (24.1–25.4)	35.1 (33.4–36.8)	27.7 (27.2–28.3)	36.6 (34.6–38.6)	31.1 (30.6–31.6)
PR (95% CI)	1.26 (1.22–1.30)^†^	—	1.38 (1.28–1.49)^†^	—	1.22 (1.14–1.31)^†^	—	1.26 (1.20–1.33)^†^	—	1.18 (1.11–1.24)^†^	—
**Binge drinking (men ≥5; women ≥4 drinks) on any occasion in past 30 days**	13.5 (12.8–14.2)	18.4 (18.1–18.7)	19.3 (17.9–20.8)	27.7 (27.0–28.4)	14.6 (13.2–16.1)	20.4 (19.8–21.0)	10.3 (9.2–11.4)	13.4 (13.0–13.8)	4.7 (3.9– 5.7)	4.6 (4.4–4.8)
PR (95% CI)	0.73 (0.69–0.77)^†^	—	0.70 (0.64–0.76)^†^	—	0.72 (0.65–0.80)^†^	—	0.77 (0.69–0.86)^†^	—	1.03 (0.85–1.25)	—
***Weight status: % (95% CI)***										
**Normal (body mass index of 18.5–24.9 kg/m^2^)**	26.0 (25.2–26.9)	34.4 (34.0–34.7)	38.9 (37.0–40.8)	44.5 (43.8–45.3)	19.9 (18.4–21.5)	30.2 (29.5–30.9)	18.6 (17.3–19.9)	27.4 (26.9–27.9)	23.8 (22.1–25.6)	32.1 (31.7–32.6)
PR (95% CI)	0.76 (0.74–0.79)^†^	—	0.87 (0.83–0.92)^†^	—	0.66 (0.61–0.71)^†^	—	0.68(0.63–0.73)^†^	—	0.74 (0.69–0.80)^†^	—
**Obesity (body mass index of ≥30 kg/m^2^)**	37.4 (36.5–38.3)	28.4 (28.1–28.7)	28.7 (27.1–30.5)	22.1 (21.5–22.8)	42.8 (40.8–44.7)	32.3 (31.6–33.0)	43.3 (41.5–45.1)	33.3 (32.8–33.8)	35.8 (33.9–37.7)	27.0 (26.5–27.4)
*PR (95% CI)*	1.31 (1.27–1.35)^†^	—	1.30 (1.22–1.39)^†^	—	1.33 (1.26–1.39)^†^	—	1.30 (1.24–1.36)^†^	—	1.33 (1.25–1.40)^†^	—
***Health care utilization: % (95% CI)***										
**Has a personal doctor or health care provider**	77.4 (76.6–78.2)	78.5 (78.2–78.8)	61.5 (59.7–63.3)	64.2 (63.5–64.9)	77.6 (75.9–79.3)	77.3 (76.7–77.9)	86.7 (85.5–87.9)	87.7 (87.3–88.1)	93.9 (92.9–94.8)	95.2 (95.5–95.4)
PR (95% CI)	0.99 (0.98–1.00)	—	0.96 (0.93–0.99)^†^	—	1.00 (0.98–1.03)	—	0.99 (0.98–1.00)	—	0.99 (0.98–1.00)	—
**Could not see doctor in past year because of cost**	17.3 (16.5–18.0)	12.6 (12.3–12.8)	19.4 (17.9–20.9)	15.1 (14.6–15.7)	18.8 (17.4–20.3)	15.0 (14.4–15.5)	17.7 (16.5–19.1)	12.3 (11.9–12.7)	10.1 (8.8–11.5)	4.2 (4.0– 4.4)
PR (95% CI)	1.37 (1.31–1.44)^†^	—	1.28 (1.17–1.39)^†^	—	1.26 (1.15–1.37)^†^	—	1.44 (1.33–1.56)^†^	—	2.41 (2.09–2.78)^†^	—
**Take antihypertensive medication to control blood pressure among adults with high blood pressure**	65.4 (63.8–67.0)	57.7 (56.9–58.4)	31.5 (27.3–36.1)	22.2 (20.4–24.1)	70.5 (67.5–73.3)	59.3 (57.8–60.7)	88.2 (86.8–89.5)	81.1 (80.4–81.8)	94.9 (94.0–95.7)	92.6 (92.3–92.9)
PR (95% CI)	1.08 (1.07–1.09)^†^	—	1.42 (1.21–1.67)^†^	—	1.19 (1.13–1.25)^†^	—	1.09 (1.07–1.11)^†^	—	1.03 (1.02–1.04)^†^	—
***Health-related quality of life: % (95% CI)***										
**Fair or poor health status**	21.7 (21.0–22.5)	15.9 (15.6–16.1)	11.9 (10.7–13.2)	9.1 (8.6– 9.5)	17.7 (16.3–19.1)	14.5 (14.0–15.1)	29.8 (28.3–31.3)	21.2 (20.7–21.7)	36.1 (34.2–38.0)	23.7 (23.3–24.2)
PR (95% CI)	1.36 (1.32–1.42)^†^	—	1.31 (1.17–1.47)^†^	—	1.22 (1.12–1.33)^†^	—	1.40 (1.33–1.48)^†^	—	1.52 (1.44–1.61)^†^	—
**Frequent mental distress (≥14 days in past 30 days)**	13.0 (12.4–13.6)	11.7 (11.4–11.9)	13.8 (12.5–15.2)	13.0 (12.5–13.6)	13.2 (12.0–14.4)	12.6 (12.2–13.1)	14.3 (13.2–15.5)	12.1 (11.7–12.4)	9.2 (8.1–10.4)	7.1 (6.8–7.4)
PR (95% CI)	1.11 (1.05–1.17)^†^	—	1.06 (0.96–1.17)	—	1.04 (0.94–1.15)	—	1.19 (1.09–1.30)^†^	—	1.30 (1.14–1.48)^†^	—
**Frequent physical distress (≥14 days in past 30 days)**	12.8 (12.3–13.5)	11.6 (11.4–11.8)	6.3 (5.4– 7.4)	6.4 (6.1– 6.8)	11.5 (10.3–12.8)	10.5 (10.1–11.0)	18.3 (17.0–19.6)	16.3 (15.9–16.8)	20.1 (18.6–21.7)	16.8 (16.4–17.2)
PR (95% CI)	1.11 (1.05–1.16)^†^	—	0.99 (0.84–1.16)	—	1.09 (0.98–1.23)	—	1.12 (1.04–1.21)^†^	—	1.20 (1.10–1.30)^†^	—
***Chronic conditions: % (95% CI)***										
**Asthma**	10.8 (10.2–11.3)	8.8 (8.6– 9.0)	11.1 (10.0–12.3)	9.4 (9.0– 9.9)	11.0 (9.9–12.2)	8.6 (8.3– 9.0)	11.0 (10.1–12.0)	8.9 (8.6– 9.2)	9.5 (8.5–10.7)	8.1 (7.8– 8.4)
PR (95% CI)	1.22 (1.15–1.29)^†^	—	1.18 (1.05–1.32)^†^	—	1.28 (1.16–1.45)^†^	—	1.24 (1.13–1.36)^†^	—	1.18 (1.05–1.33)^†^	—
**Chronic obstructive pulmonary disease**	5.9 (5.5–6.3)	6.1 (6.0– 6.3)	2.9 (2.4–3.5)	2.4 (2.2–2.7)	4.4 (3.8–5.1)	4.0 (3.7–4.2)	8.2 (7.4–9.0)	9.0 (8.7–9.3)	11.0 (9.8–12.2)	12.9 (12.6–13.3)
PR (95% CI)	0.96 (0.90–1.03)	—	1.23 (1.00–1.52)	—	1.11 (0.94–1.31)	—	0.91 (0.82–1.00)	—	0.85 (0.76–0.95)^†^	—
**High blood pressure**	40.3 (39.5–41.1)	28.9 (28.6–29.2)	12.0 (11.0–13.2)	9.9 (9.4–10.3)	32.7 (31.0–34.5)	21.9 (21.3–22.5)	61.2 (59.6–62.9)	40.8 (40.3–41.4)	77.0 (75.1–78.8)	60.3 (59.8–60.8)
PR (95% CI)	1.37 (1.34–1.40)^†^	—	1.22 (1.10–1.35)^†^	—	1.49 (1.41–1.59)^†^	—	1.50 (1.45–1.55)^†^	—	1.28 (1.25–1.31)^†^	—
**High blood cholesterol**	31.3 (30.4–32.2)	31.5 (31.2–31.8)	11.5 (10.1–13.2)	13.1 (12.4–13.7)	26.7 (24.9–28.6)	28.2 (27.5–28.9)	47.5 (45.7–49.3)	44.9 (44.3–45.5)	54.3 (52.2–56.3)	54.9 (54.4–55.4)
PR (95% CI)	1.00 (0.97–1.02)	—	0.88 (0.76–1.02)	—	0.95 (0.88–1.02)	—	1.06 (1.02–1.10)^†^	—	0.99 (0.95–1.03)	—
**Diabetes**	14.4 (13.9–15.0)	8.7 (8.6–8.9)	1.5 (1.2–1.8)	1.4 (1.2–1.5)	9.7 (8.6–10.9)	5.8 (5.4– 6.1)	23.1 (21.7–24.6)	13.7 (13.3–14.1)	34.9 (33.0–36.8)	20.8 (20.3–21.2)
PR (95% CI)	1.64 (1.57–1.71)^†^	—	1.06 (0.82–1.38)	—	1.68 (1.48–1.92)^†^	—	1.69 (1.58–1.81)^†^	—	1.68 (1.59–1.78)^†^	—
**Coronary heart disease (including history of heart attack or angina)**	6.0 (5.6–6.4)	5.7 (5.6–5.8)	0.7 (0.5–1.1)	0.7 (0.6–0.9)	3.1 (2.6–3.7)	2.5 (2.3–2.7)	9.8 (8.8–11.1)	7.8 (7.5–8.1)	15.7 (14.3–17.3)	17.6 (17.2–18.0)
PR (95% CI)	1.06 (0.99–1.14)	—	1.00 (0.67–1.51)	—	1.23 (1.00–1.51)	—	1.26 (1.13–1.42)^†^	—	0.89 (0.81–0.98)^†^	—
**Stroke**	4.1 (3.8–4.4)	2.6 (2.5–2.7)	0.7 (0.5–1.1)	0.4 (0.4–0.5)	2.4 (1.9– 2.9)	1.3 (1.2–1.5)	6.8 (6.0–7.7)	3.5 (3.3–3.7)	9.6 (8.5–10.8)	7.4 (7.2– 7.7)
PR (95% CI)	1.60 (1.47–1.75)^†^	—	1.73 (1.09–2.75)^†^	—	1.77 (1.39–2.24)^†^	—	1.95 (1.70–2.23)^†^	—	1.29 (1.14–1.46)^†^	—
**Cancer (excluding skin cancer)**	5.1 (4.8–5.5)	6.5 (6.3–6.6)	0.9 (0.6–1.3)	1.5 (1.4–1.7)	2.3 (1.8–2.9)	3.4 (3.2–3.7)	6.6 (5.9–7.4)	8.1 (7.8–8.4)	15.8 (14.4–17.2)	18.4 (18.0–18.8)
PR (95% CI)	0.80 (0.74–0.86)^†^	—	0.58 (0.38–0.89)^†^	—	0.67 (0.53–0.86)^†^	—	0.82 (0.72–0.92)^†^	—	0.86 (0.78–0.94)^†^	—

**Abbreviation:** CI = confidence interval.

* Percentages for adults aged ≥18 years were age-standardized to the U.S. 2000 population aged ≥18 years.

^†^ Statistical significance at alpha = 0.05; 95% CI did not include 1.0.

In all age groups, blacks were more likely than whites to report not being able to see a doctor in the past year because of cost. Blacks aged 18–34 years were less likely to have a personal doctor or health care provider than whites (Table 3). Blacks with high blood pressure were more likely than whites in each age group to report taking medication to control it.

Blacks in all age groups were more likely to report fair to poor health status than whites (Table 3). Blacks were more likely than whites to report frequent mental distress and frequent physical distress at age ≥50 years. The prevalence of having diagnoses of some chronic conditions was higher among blacks than whites across age groups, including for asthma, high blood pressure, diabetes, and stroke. In contrast, blacks across all age groups were less likely than whites to report a cancer diagnosis.

In 2015, blacks had 40% higher death rates than whites for all-cause mortality in all age groups <65 years (Table 4). At ages 18–34 years, blacks had higher death rates than whites for eight of the 10 leading causes of death among blacks in that age group (heart disease; cancer; cerebrovascular disease; diabetes mellitus; homicide; HIV disease; and conditions resulting from pregnancy, childbirth, and the puerperium). At ages 35–49 years, blacks had higher death rates than whites for heart disease; cancer; cerebrovascular disease; diabetes mellitus; homicide; nephritis, nephrotic syndrome, and nephrosis; septicemia; and HIV disease. At ages 50–64 years, blacks had higher death rates than whites for leading chronic diseases (heart disease, cancer; cerebrovascular disease; diabetes mellitus; and nephritis, nephrotic syndrome, and nephrosis) as well as for unintentional injury, septicemia, and HIV disease. Death rates from heart disease, cancer, cerebrovascular disease, diabetes mellitus, and homicide began increasing at earlier ages among blacks than among whites. There were significant declines in deaths from HIV disease in the past 17 years for both racial populations. Among persons aged 35–49 years, there were 45 fewer HIV disease deaths per 100,000 among blacks during 2015 compared with 1999, while among whites there were six fewer HIV disease deaths (Table 1). However, during 2015, blacks in age groups 18–34, 35–49, and 50–64 were seven to nine times more likely than whites to die from HIV disease. Some age groups of blacks had lower death rates than whites for four leading causes of death: ages 18–49 years for unintentional injuries, ages 50–64 years for chronic liver disease and cirrhosis, ages ≤49 years for suicide, and ages ≥65 years for Alzheimer’s disease.

**TABLE 4:** Comparison of death rates* for selected leading causes of death (ranked by rate) among blacks and whites, by age group and rate ratio (RR) — United States, 2015

Leading causes of death^†^	All ages*		18–34 years		35–49 years		50–64 years		≥65 years	
	Black	White	Black	White	Black	White	Black	White	Black	White
**U.S. resident population (no. in millions)**	**44.9**	**251.9**	**11.8**	**56.8**	**8.5**	**47.5**	**7.8**	**51.3**	**4.4**	**40.8**
**All causes**	**851.9 (848.9–855.0)**	**735.0 (734.1–736.0)**	**141.5 (139.4–143.7)**	**100.3 (99.5–101.1)**	**311.5 (307.7–315.2)**	**220.3 (218.9–221.6)**	**1,046.0 (1,038.8–1,053.2)**	**722.4 (720.1–724.8)**	**4,176.0 (4,156.9–4,195.0)**	**4,286.9 (4,280.6–4,293.3)**
**RR (95% CI)**	**1.16 (1.15–1.16)^§^**	—	**1.41 (1.39–1.44)^§^**	—	**1.41 (1.40–1.43)^§^**	—	**1.45 (1.44–1.46)^§^**	—	**0.97 (0.97–0.98)^§^**	—
**1. Diseases of the heart (also 1 among whites)**	205.1 (203.5–206.6)	167.9 (167.4–168.3)	10.7 (10.1–11.3)	5.1 (4.9–5.3)	66.5 (64.8–68.3)	33.1 (32.8–33.8)	257.5 (254.0–261.1)	148.1 (147.0–149.1)	1,085.5 (1,075.8–1,095.2)	1,091.8 (1,088.6–1,095.0)
RR (95% CI)	1.22 (1.21–1.23)^§^	—	2.10 (1.97–2.25)^§^	—	2.00 (1.94–2.06) ^§^	—	1.74 (1.71–1.78)^§^	—	0.99 (0.98–1.00)	—
**2. Malignant neoplasms (2)**	180.1 (178.8–181.5)	159.4 (159.0–160.0)	7.3 (6.8–7.8)	6.4 (6.2–6.7)	49.2 (47.7–50.7)	39.9 (39.4–40.5)	296.2 (292.4–300.0)	227.6 (226.3–228.9)	927.7 (918.7–936.7)	909.6 (906.6–912.5)
RR (95% CI)	1.13 (1.12–1.14)^§^	—	1.13 (1.05–1.22)^§^	—	1.23 (1.19–1.27)^§^	—	1.30 (1.28–1.32)^§^	—	1.04 (1.03–1.05)^§^	—
**3. Cerebrovascular diseases (5)**	50.8 (50.1–51.6)	36.4 (36.2–36.6)	1.6 (1.4–1.8)	0.8 (0.8–0.9)	12.8 (12.0–13.5)	4.9 (4.7–5.1)	50.5 (48.9–52.1)	21.0 (20.6–21.4)	287.2 (282.2–292.2)	245.7 (244.2–247.3)
RR (95% CI)	1.40 (1.38–1.42)^§^	—	1.94 (1.64–2.30)^§^	—	2.60 (2.42–2.79)^§^	—	2.40 (2.32–2.49)^§^	—	1.14 (1.12–1.16)^§^	—
**4. Unintentional injury (4)**	36.8 (36.3–37.4)	46.0 (45.8–46.3)	30.8 (29.8–31.8)	45.4 (44.9–46.0)	39.5 (38.2–40.9)	49.1 (48.5–49.8)	55.5 (53.8–57.1)	50.5 (49.9–51.1)	—	—
RR (95% CI)	0.80 (0.79–0.81)^§^	—	0.68 (0.66–0.70)^§^	—	0.80 (0.78–0.83)^§^	—	1.10 (1.06–1.13)^§^	—	—	—
**5. Diabetes mellitus (7)**	37.0 (36.3–37.6)	19.6 (19.4–19.8)	2.7 (2.4–3.0)	1.1 (1.0–1.2)	13.1 (12.4–13.9)	6.2 (5.9–6.4)	51.8 (50.2–53.4)	25.9 (25.5–26.3)	189.4 (185.3–193.4)	109.9 (108.8–110.9)
RR (95% CI)	1.89 (1.85–1.92)^§^	—	2.45 (2.14–2.80)^§^	—	2.13 (1.99–2.29)^§^	—	2.00 (1.93–2.07)^§^	—	1.72 (1.68–1.76)^§^	—
**6. Chronic lower respiratory disease (3)**	28.9 (28.4–29.5)	44.5 (44.2–44.7)	—	—	—	—	30.5 (29.3–31.7)	35.1 (34.6–35.6)	167.0 (163.2–170.8)	291.1 (289.4–292.8)
RR (95% CI)	0.65 (0.64–0.66)^§^	—	—	—	—	—	0.87 (0.83–0.91)^§^	—	0.58 (0.56–0.59)^§^	—
**7. Homicide (18)**	19.8 (19.4–20.2)	3.3 (3.2–3.3)	47.2 (46.0–48.5)	5.5 (5.3–5.7)	21.9 (20.9–22.8)	4.2 (4.0–4.3)	—	—	—	—
RR (95% CI)	6.04 (5.86–6.23)^§^	—	8.59 (8.22–8.97)^§^	—	5.27 (4.94–5.61)^§^	—	—	—	—	—
**8. Nephritis, nephrotic syndrome, and nephrosis (10)**	25.4 (24.9–26.0)	12.2 (12.1–12.3)	—	—	6.5 (6.0–7.0)	1.8 (1.7–1.9)	27.1 (26.0–28.3)	8.6 (8.4–8.9)	143.3 (139.7–146.8)	82.5 (81.6–83.4)
RR (95% CI)	2.08 (2.04–2.12)^§^	—	—	—	3.63 (3.26–4.04)^§^	—	3.15 (2.99–3.32)^§^	—	1.74 (1.70–1.79)^§^	—
**9. Alzheimer’s disease (6)**	26.6 (26.1–27.2)	30.5 (30.3–30.7)	—	—	—	—	—	—	181.7 (177.7–185.7)	212.2 (210.7–213.6)
RR (95% CI)	0.87 (0.85–0.89)^§^	—	—	—	—	—	—	—	0.75 (0.73–0.77)^§^	—
**10. Septicemia (12)**	18.1 (17.6–18.5)	10.4 (10.3–10.5)	—	—	5.4 (4.9–5.9)	2.6 (2.4–2.5)	21.8 (20.8–22.9)	10.7 (10.4–10.9)	95.8 (92.9–98.7)	61.3 (60.5–62.0)
RR (95% CI)	1.74 (1.69–1.78)^§^	—	—	—	2.12 (1.91–2.36)^§^	—	2.05 (1.94–2.16)^§^	—	1.52 (1.47–1.57)^§^	—
**11.Essential hypertension and hypertensive renal disease (14)**	—	—	—	—	—	—	—	—	89.4 (86.6–92.2)	51.0 (50.3–51.7)
RR (95% CI)	—	—	—	—	—	—	—	—	1.70 (1.65–1.76)^§^	—
**12. Influenza and pneumonia (8)**	—	—	—	—	—	—	—	—	89.4 (86.7–92.2)	98.9 (97.9–99.8)
RR (95% CI)	—	—	—	—	—	—	—	—	0.86 (0.83–0.89)^§^	—
**14. HIV disease (27)**	—	—	3.4 (3.1–3.7)	0.4 (0.3–0.4)	12.2 (11.5–13.0)	1.8 (1.7–1.9)	19.7 (18.7–20.6)	2.7 (2.6–2.9)	—	—
RR (95% CI)	—	—	9.39 (7.94–11.10)^§^	—	6.90 (6.30–7.56)^§^	—	7.26 (6.75–7.80)^§^	—	—	—
**15. Chronic liver disease and cirrhosis (11)**	—	—	—	—	—	—	23.1 (22.1–24.2)	32.0 (31.5–32.5)	—	—
RR (95% CI)	—	—	—	—	—	—	0.72 (0.69–0.76)^§^	—	—	—
**16. Suicide (9)**	—	—	9.5 (8.9–10.0)	16.8 (16.5–17.1)	7.5 (6.9–8.1)	20.8 (20.4–21.2)	—	—	—	—
RR (95% CI)	—	—	0.56 (0.53–0.60)^§^	—	0.36 (0.33–0.39)^§^	—	—	—	—	—
**22. Anemias (25)**	—	—	1.5 (1.3–1.7)	—^¶^	—	—	—	—	—	—
**28. Pregnancy, childbirth, and the puerperium (31)**	—	—	1.6 (1.4–1.9)	0.6 (0.5–0.7)	—	—	—	—	—	—
RR (95% CI)	—	—	2.63 (2.21–3.13)^§^	—	—	—	—	—	—	—

**Abbreviations:** CI = confidence interval; HIV = human immunodeficiency virus; RR = rate ratio comparing death rates for blacks to that for whites.

* Rates (per 100,000 population) are provided for both blacks and whites. Overall death rates (per 100,000 population) for all ages including infants and children are age-standardized to the U.S. 2000 projected population.

^†^ Presented in rank order for the total black U.S. population (all ages) based on total numbers of death in 2015 with rank order for the total white U.S. population in parentheses. Age-specific data are provided for both race groups only for the top 10 leading causes of death among the black U.S. population in each age group.

^§^ Statistical significance at alpha = 0.05; 95% CI did not include 1.0.

^¶^ Unstable death rate for whites aged 18–49 years because there were only 35 deaths from anemias in this subgroup in 2015.

## Conclusions and Comment

During 1999–2015, age-adjusted death rates decreased by 25% for blacks and 14% for whites, with 284 fewer age-adjusted deaths per 100,000 blacks and 120 fewer age-adjusted deaths per 100,000 whites during 2015 compared with 1999. Among persons aged ≥65 years, there was a black-white mortality crossover, whereby blacks had slightly lower age-adjusted deaths than whites beginning in 2010. In addition, during 1999–2015, blacks saw declines in the two leading causes of death, heart disease and cancer, across all age groups. However, despite substantive reductions in death rates among blacks in the United States, blacks continue to have higher death rates overall, higher prevalence of many chronic health conditions, and lower prevalence of some healthy behaviors. Blacks were less likely to participate in leisure-time physical activity and maintain a healthy weight. Blacks were more likely to report not being able to see a doctor because of cost, even though, across age groups, the percentages of blacks and whites who reported having a personal doctor or health care provider were approximately equal.

In addition, this analysis shows that blacks had significantly lower educational attainment and home ownership and almost twice the proportion of households living below the poverty level and unemployed than whites in all age groups. Such social factors are posited as “fundamental causes” because they influence chronic conditions, related behaviors, health-related quality of life, and health care utilization by constraining persons’ abilities to engage in prevention or treatment (*7*,*8*). These differences in “fundamental causes,” health behaviors, and access to health care contribute to the excess deaths and chronic conditions among younger black adults that are most common among persons aged ≥65 years. For example, blacks in age groups 18–34 and 35–49 were nearly twice as likely to die from heart disease, stroke, and diabetes as whites. These findings are generally consistent with previous reports that use the term “weathering” to suggest that blacks experience premature aging and earlier health decline than whites, and that this decline in health accumulates across the entire life span and potentially across generations, as a consequence of psychosocial, economic, and environmental stressors (*9*,*10*).

Taken in the context of other research, the substantial differences in mortality, health behaviors, access to health care, and social factors across the life span identified in this analysis highlight the importance of a dual strategy of universal and targeted interventions to address disparities in black health (*11*). Opportunities for interventions have been identified that decision-makers, public health programs, clinicians, and communities can use. The Community Preventive Services Task Force has recommendations for interventions with proven effectiveness for the prevention of obesity, physical inactivity, tobacco use, promotion of cancer screening, and medication adherence (https://www.thecommunityguide.org/). CDC has also released a series of violence prevention technical packages to help communities use the strategies with the best available evidence (https://www.cdc.gov/violenceprevention/pub/technical-packages.html). To ensure continued progress in improving health for all U.S. residents, targeted interventions for populations living in vulnerable social and economic conditions (e.g., poverty or racially segregated neighborhoods with fewer resources) also should be considered. The U.S. Department of Health and Human Services Action Plan to Reduce Racial and Ethnic Health Disparities promotes targeted interventions to reduce these disparities (https://www.minorityhealth.hhs.gov/npa/files/Plans/HHS/HHS_Plan_complete.pdf). In addition, The Racial and Ethnic Approaches to Community Health (REACH) program, which supports targeted interventions through community-based, participatory approaches, identified strategies to address health disparities for blacks and other racial/ethnic populations (*12*–*15*).

The findings in this report are subject to at least six limitations. First, information about many characteristics were self-reported and subject to recall and social desirability biases, although this is unlikely to account for large disparities within the analyses (*16*). Second, this was a cross-sectional analysis, and data do not allow a comparison of rates for the same cohort as they aged (*16*). Third, the American Community Survey and BRFSS are household surveys and exclude persons living in institutions, long-term care facilities, and prisons. Fourth, there are technical and conceptual limitations associated with examining race in epidemiologic analyses because it is complex and generally represents other economic, psychosocial, and environmental factors (*17*–*19*). Fifth, although whites were considered as the benchmark (*20*), or referent in this analysis, blacks had lower death rates for unintentional injury and suicide in some age groups and lower prevalences of binge drinking. Finally, differences within blacks and whites by sex, socioeconomic characteristics, and Hispanic subgroups were not considered, yet might modulate some of the relationships seen overall.

Optimizing health for all U.S. residents, while also eliminating disparities, remains an integral part of disease prevention and health promotion activities. Although significant strides have been made in the United States in the last 17 years, disparities still exist. To continue to improve the health of the black population, there is a continued need to translate research results into effective universal and targeted interventions across the lifespan to inform action.

**Key Points**

• In the United States, there were fewer age-adjusted deaths per 100,000 during 2015 compared with 1999, with 284 fewer among blacks and 120 fewer among whites.

• Despite the narrowing of disparities in death rate for blacks and whites, disparities in the leading causes of deaths for blacks compared with whites remain large and persistent across the life span. Blacks had higher death rates than whites for all-cause mortality in all age groups <65 years.

• Blacks had significantly lower educational attainment and home ownership and almost twice the proportion of households below the poverty level compared with whites across the life span. This might help explain disparities in mortality via chronic disease–related behaviors, health-related quality of life, and health care utilization.

• Universal and targeted interventions are needed to reduce black-white health disparities across the life span.

• Additional information is available at https://www.cdc.gov/vitalsigns.

## Footnotes

* https://wonder.cdc.gov.

† Leading causes of death are defined by *International Classification of Diseases, Tenth Revision* (ICD-10) codes for diseases that are reported as the underlying cause of death on the death certificate. Among blacks in 2015, the 10 leading causes of death for ages 18–34 years were 1) homicide (U01–U02, X85–Y09, Y87.1); 2) unintentional injury (V01–X59, Y85–Y86), 3) diseases of heart (I00–I09, I11, I13, I20–I51); 4) suicide (U03, X60–X84, Y87.0); 5) malignant neoplasms (C00–C997); 6) human immunodeficiency virus (HIV) disease (B20–B24), 7) diabetes mellitus (E10–E14); 8) pregnancy, childbirth and the puerperium (O00–O99); 9) cerebrovascular disease (I60–I69); and 10) anemias (D50–D64) . For ages 35–49 years the leading causes were 1) diseases of heart; 2) malignant neoplasms; 3) unintentional injury; 4) homicide; 5) diabetes mellitus; 6) cerebrovascular disease; 7) HIV disease; 8) suicide; 9) nephritis, nephrotic syndrome and nephrosis (N00–N07, N17–N19, N25–N27); and 10) septicemia (A40–A41). For ages 50–64 years the leading causes were 1) malignant neoplasms; 2) diseases of heart; 3) unintentional injury; 4) diabetes mellitus; 5) cerebrovascular disease; 6) chronic lower respiratory disease (J40–J47); 7) nephritis, nephrotic syndrome and nephrosis; 8) chronic liver disease and cirrhosis (K70, K73–K74); 9) septicemia; and 10) HIV disease. For ages ≥65 years the leading causes were 1) diseases of heart; 2) malignant neoplasms; 3) cerebrovascular disease; 4) diabetes mellitus; 5) Alzheimer’s disease (G30); 6) chronic lower respiratory disease; 7) nephritis, nephrotic syndrome and nephrosis; 8) septicemia; 9) influenza and pneumonia (J09–J18); and 10) essential hypertension and hypertensive renal disease (I10, I12, I15).

§ https://www.census.gov/programs-surveys/popest.html.

¶
https://www.census.gov/programs-surveys/acs/data/pums.html .

** https://www.cdc.gov/brfss/annual_data/annual_2015.html.
