# Erratum: Vol. 66, No. 17

**DOI:** 10.15585/mmwr.mm6618a11

**Published:** 2017-05-12

In the report “Vital Signs: Racial Disparities in Age-Specific Mortality Among Blacks or African Americans — United States, 1999–2015,” on page 446, under “Results,” the last sentence of the first paragraph should have read “Among adults aged ≥65 years, the death rate in 2015 relative to that in 1999 declined 27% for blacks and 17% for whites, resulting in a crossover in death rates **after 2010, when blacks had lower age-specific death rates** than whites.”

On page 447, the first sentence of the first paragraph should have read, “Among persons aged ≥65 years, there was a black-white mortality crossover, whereby blacks had slightly lower **age-specific death rates than whites after 2010**.”

